# Molecularly Imprinted Electropolymer for a Hexameric Heme Protein with Direct Electron Transfer and Peroxide Electrocatalysis

**DOI:** 10.3390/s16030272

**Published:** 2016-02-23

**Authors:** Lei Peng, Aysu Yarman, Katharina J. Jetzschmann, Jae-Hun Jeoung, Daniel Schad, Holger Dobbek, Ulla Wollenberger, Frieder W. Scheller

**Affiliations:** 1 Institute of Biochemistry and Biology, Potsdam University, Karl-Liebknecht-Strasse 24-25, 14476 Potsdam, Germany; penglei0525@163.com (L.P.); aysu.yarman@yahoo.de (A.Y.); jetzschm@uni-potsdam.de (K.J.J.); uwollen@uni-potsdam.de (U.W.); 2 Institute of Biology, Structural Biology/Biochemistry, Humboldt-Universität zu Berlin, Unter den Linden 6, 10099 Berlin, Germany; jae-hun.jeoung@biologie.hu-berlin.de (J.-H.J.); schad.daniel@googlemail.com (D.S.); holger.dobbek@biologie.hu-berlin.de (H.D.); 3 Fraunhofer Institute for Cell Therapy and Immunology IZI-BB, Am Mühlenberg 13, 14476 Potsdam, Germany

**Keywords:** molecularly imprinted polymers, self-assembled monolayer, direct electron transfer, hydrogen peroxide, bioelectrocatalysis

## Abstract

For the first time a molecularly imprinted polymer (MIP) with direct electron transfer (DET) and bioelectrocatalytic activity of the target protein is presented. Thin films of MIPs for the recognition of a hexameric tyrosine-coordinated heme protein (HTHP) have been prepared by electropolymerization of scopoletin after oriented assembly of HTHP on a self-assembled monolayer (SAM) of mercaptoundecanoic acid (MUA) on gold electrodes. Cavities which should resemble the shape and size of HTHP were formed by template removal. Rebinding of the target protein sums up the recognition by non-covalent interactions between the protein and the MIP with the electrostatic attraction of the protein by the SAM. HTHP bound to the MIP exhibits quasi-reversible DET which is reflected by a pair of well pronounced redox peaks in the cyclic voltammograms (CVs) with a formal potential of −184.4 ± 13.7 mV *vs.* Ag/AgCl (1 M KCl) at pH 8.0 and it was able to catalyze the cathodic reduction of peroxide. At saturation the MIP films show a 12-fold higher electroactive surface concentration of HTHP than the non-imprinted polymer (NIP).

## 1. Introduction

MIPs gain attention as artificial recognition elements both in scientific and industrial fields [1,2,3]. Molecular imprinting can be described as the formation of specific recognition sites in a material by polymerizing a monomer in the presence of a target molecule, the so-called template. During this process, functional monomers interact with complementary functional groups of the template and this complex is trapped in a polymeric matrix. After the template’s removal by washing or extraction, cavities with specific size, shape, and functionalities are formed which recognize the template during the rebinding process [4].

Small molecules like sugars, steroids, drugs, and amino acid derivatives [5] have been successfully imprinted. Despite the importance of generating effective synthetic receptors for biomacromolecules less than 2% of the MIP literature is on macromolecular imprinting and only a few papers have described MIPs for enzymes [6,7,8,9,10,11,12,13,14,15]. Imprinting of biomacromolecular targets, especially proteins, is still a challenging task due to their large sizes, conformational flexibility, and instability in organic solvents [16].

Preparation of MIPs by electropolymerization circumvents several of these problems: electropolymerization makes it possible to optimize the film thickness in order to generate binding sites accessible to the target. Furthermore, the MIP formation proceeds directly on the transducer surface and can frequently be carried out in aqueous environments. These features are important for protein MIPs that require mild preparation procedures [17,18].

Recently we prepared an electrosynthesized MIP on top of a negatively charged SAM for the electron carrier protein cytochrome *c* (cyt *c*), where the oriented assembly of the protein on the negatively charged surfaces has been exploited to facilitate DET at the electrode [19]. Up to now bioelectrocatalysis for a protein MIP has not yet been reported in the literature. In this paper we describe a MIP with both DET and bioelectrocatalysis for a cosubstrate. This is demonstrated for the hexameric enzyme HTHP which exhibits an intrinsic peroxidatic activity. The bioelectrocatalytic enhancement of peroxide reduction gives evidence of both the productive binding inside the MIP cavities and the retained activity of the MIP-bound enzyme. Furthermore, the SAM/MIP architecture shows a preferential binding to its target in respect to cyt *c*, which otherwise strongly binds to the negatively charged (non-imprinted) SAM.

## 2. Materials and Methods

### 2.1. Chemicals

Hydrogen peroxide (H_2_O_2_ 30%), [Ru(NH_3_)_6_]Cl_2_, 11-mercaptoundecanoic acid (MUA), cyt *c* (from equine heart, MW = 12,384 Da), and scopoletin (7-hydroxy-6-methoxycoumarin) were bought from Sigma-Aldrich (Steinheim, Germany). NADH was bought from Gerbu Biotechnic GmbH (Germany). All other reagents were of analytical grade and used without further purification.

HTHP, (pI 5.6), was prepared as described by Jeoung *et al.* [20]. It was expressed in *Escherichia coli Rosetta* (DE3) by induction with 1 μM isopropyl-ß-d-thiogalactopyranoside. Approximately 50 mg HTHP per liter of culture were purified to homogeneity as determined by a single band of approximately 7.9 kDa (for the monomeric form) on SDS-PAGE using standard protein purification protocols. Purified HTHP showed a R.Z. value of 0.26 (ratio of absorbance at Soret peak and 280 nm), which was increased to 2.8 by reconstitution of this preparation with equal molar hemin chloride. The final concentration of the purified hexameric HTHP was 1.3 mM in 50 mM Tris-HCl at pH 8 with 150 mM NaCl.

### 2.2. Preparation of Electrodes

Gold wire electrodes with a diameter of 0.5 mm and an active area of 0.161 cm^2^ from Goodfellow, Germany, were boiled in 2 M KOH solution for 4 h and kept in concentrated HNO_3_ for 10 min. After careful rinsing with Millipore water, they were stored in concentrated H_2_SO_4_ when not in use. Before every usage, the electrodes were washed with Millipore water and kept in concentrated HNO_3_ for 10 min, then rinsed by Millipore water again in each successive step.

The cleaned electrodes were incubated in 5 mM MUA at least overnight at 4 °C. MUA was dissolved in 96 % ethanol and freshly prepared each time before modification. After being washed in Millipore water, a MUA modified gold wire electrode was immersed directly in 1.3 mM HTHP solution for 3 h at 4 °C to get the HTHP loaded electrode.

For the preparation of the HTHP-MIP polyscopoletin was deposited on the MUA covered Au electrode by electropolymerization from an aqueous solution of 0.5 mM scopoletin and 5 mM NaCl. A single potential pulse of 0.7 V for 5 s was followed by 0 V for 5 s. After formation of the HTHP-MIP, the modified electrodes were rinsed with water. The template protein, HTHP, was removed by incubating the HTHP-MIPs in 50 mM glycine-HCl (pH 2.2) on a shaker at 300 rpm for 1 h (25 °C). After template removal, the MIPs were rinsed with water and and could be stored in 2.5 mM phosphate buffer (PB) at pH 7 for one week. NIPs were prepared in the same manner but in absence of the protein and incubated in 50 mM glycine-HCl (pH 2.2) before the measurements.

### 2.3. Apparatus and Electrochemical Measurements

Electrochemical measurements were carried out with a PalmSens (Utrecht, Netherlands) electrochemical station. A three-electrode system with a working electrode, a Pt wire as the counter electrode and an Ag/AgCl (1 M KCl) as the reference electrode was used in all electrochemical experiments. Both CV and square wave voltammetry (SWV) were conducted in a 2 mL compartment of a custom-built reaction chamber with an adjustable magnetic stirring system. All experiments were performed at room temperature (25 °C) with exclusion of oxygen.

The DET of HTHP-MIPs was recorded by CV in 2.5 mM Tris buffer or PB at pH 8 from −0.5 to 0.2 V at different scan rates. The permeability of the MIP layer for the redox marker [Ru(NH_3_)_6_]^2+^ after electropolymerization, template removal and rebinding was characterized by CVs between −500 and 0 mV in 100 mM NaCl at a scan rate of 100 mV/s. The concentration dependence of HTHP rebinding to the MIPs were performed after 1 h of incubation in HTHP-containing solutions in 2.5 mM PB at pH 7 by CV and SWV.

Bioelectrocatalytic reduction of H_2_O_2_ was investigated by CV in 2.5 mM Tris buffer at pH 8 (25 °C) with exclusion of oxygen. After each addition of H_2_O_2_ to the working buffer, CVs were recorded at a scan rate of 5 mV/s after 2 min of reaction time. NADH oxidation by HTHP MIPs was studied in the presence of 10 µM H_2_O_2_, aliquots of NADH were injected into the working cell and CVs were recorded at a scan rate of 5 mV/s after 2 min of reaction time.

## 3. Results and Discussion

### 3.1. Preparation of the MIP-Modified Electrode, Template Removal and Rebinding

**Figure 1:**
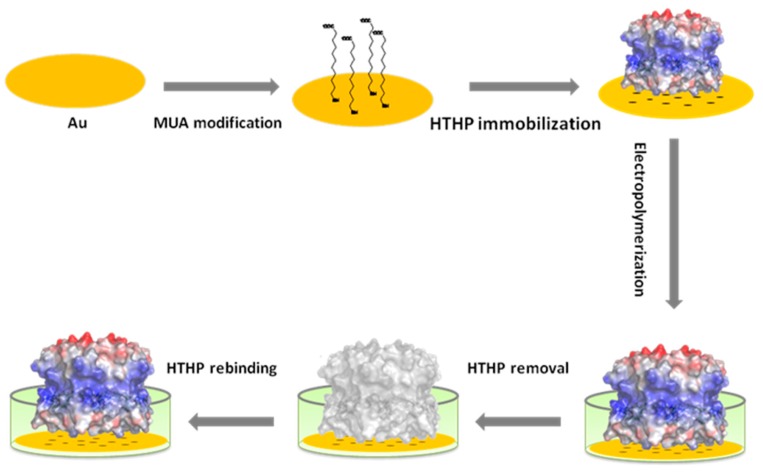
Schematic representation of the MIP preparation on a negatively charged thiol terminated SAM; Red: negatively charged region of HTHP, blue: positive region.

The MUA-modified electrode which is loaded with HTHP showed a pair of well pronounced peaks in the CV (Figure S1). For the electrochemical MIP preparation it was inserted in 0.5 mM scopoletin solution containing 5 mM NaCl. The low ionic strength was used in order to prevent the dissociation of HTHP from the SAM. The electrode was polarized for 5 s at 0.7 V followed by 5 s at 0 V. In this process scopoletin is polymerized and forms a network around the template HTHP (Figure 1). Scopoletin was chosen as the monomer [21] because in previous work we succeeded to prepare MIPs for cyt *c* [19] and the lectin concanavalin A (ConA) [22] by electropolymerizing it on top of a SAM.

**Figure 2:**
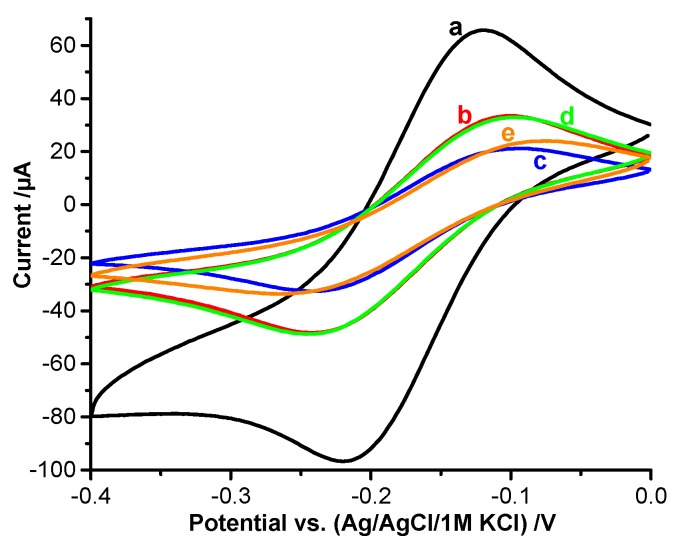
CVs of the redox marker [Ru(NH_3_)_6_]^2+^ for the different steps of MIP preparation (5 mM [Ru(NH_3_)_6_]^2+^ in 100 mM NaCl, pH 8, 100 mV/s): a—bare Au wire, b—after SAM-formation, c—after electropolymerization in presence of the template HTHP, d—after removal of HTHP, e—after rebinding in 1.3 mM HTHP solution for 1 h.

In order to characterize each step of the MIP-preparation, we studied the diffusive permeability of redox markers and the DET of the heme prosthetic groups by CVs. Whilst the redox peaks of the negatively charged ferricyanide are completely suppressed by the MUA-SAM, the peak currents of the positively charged [Ru(NH_3_)_6_]^2+^ are almost 40 percent of the currents of the bare electrode (Figure 2b). Formation of the MIP layer by electropolymerization on top of the HTHP loaded MUA-SAM brought about a further decrease of the peaks of [Ru(NH_3_)_6_]^2+^ by almost 55 percent (Figure 2c and Figure S2). Treatment of the MIP electrode with glycine-HCl resulted in a marked increase of the peak currents (Figure 2d and Figure S2) which indicates the formation of “diffusion pathways” for the small redox marker by the removal of the template. After incubation in HTHP containing solution (Figure 2e) the peak currents decreased to the values after electropolymerization. Parallel experiments showed that the peaks of the DET of the heme groups of HTHP were decreased by electropolymerization. Treatment of the HTHP-MIP with glycine-HCl brought about an almost complete depression of the DET signal of HTHP (Figure S3). Rebinding of the target resulted in the reestablishment of the DET peaks. Both of the results with the redox marker, and for the DET, indicate that the target HTHP was effectively removed by the treatment with glycine-HCl and afterwards rebound from the HTHP containing solution.

Under comparable conditions of electropolymerization, the thickness of the scopoletin-based MIP containing ConA was estimated by atomic force microscopy (AFM) and surface plasmon resonance (SPR) [22] to be 3.2 nm. This thickness of the polyscopoletin layer is comparable to the dimension of the HTHP molecule (17.3 nm × 17.3 nm × 4.59 nm) [20] which may explain the effective template removal.

### 3.2. DET of HTHP Trapped in the MIP

HTHP from *Silicibacter pomeroyi* has a hexameric ring structure with a molecular mass of about 54 kDa. The six monomers are equivalent and contain one non-covalently bound heme in a hydrophobic pocket. The iron is coordinated by tyrosine in the proximal side while the distal side is encompassed by arginine [20]. The isoelectric point of HTHP is 5.6, so the overall charge at pH 8 should be negative. Theoretical calculations show that, on negatively charged surfaces, the HTHP disc binds to the surface via its neutrally charged side (Figure 1). In this “disc model”, the distance between the six heme groups and the surfaces is shortest hence an efficient electron transfer could be expected [23].

HTHP adsorbed on MUA modified electrodes (HTHP-MUA-Au) displayed a pair of redox peaks with peak potentials at −165 and −251 mV. These redox peaks are typical for the Fe^2+^/Fe^3+^ couple of heme containing proteins [24]. The appearance of only one peak pair and its shape is in accordance with an electrode reaction of a multi-redox center species with non- or weakly interacting centers [25].

**Figure 3:**
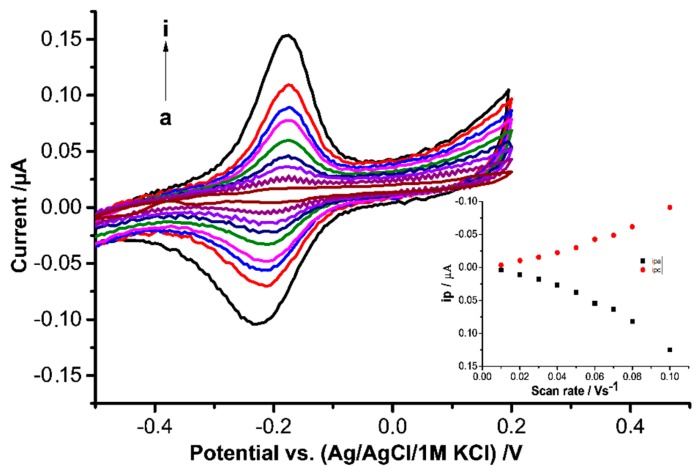
CVs at different scan rates for HTHP-MIP (after rebinding in 1.3 mM HTHP solution), a—i: 10, 20, 30, 40, 50, 60, 70, 80, 90, 100 mV/s in 2.5 mM Tris buffer, pH 8. Inset: Dependence of peak currents on scan rates.

After the formations of the MIP by electropolymerization on top of the HTHP loaded MUA-SAM the pair of the remaining peaks possessed a formal potential of −184.4 ± 13.7 mV *vs.* Ag/AgCl 1 M KCl at pH 8.0 (Figure 3). Compared to the value for HTHP at MUA modified electrode [23], it is only slightly positively shifted, which indicates that HTHP was not greatly altered by electropolymerization. 

As shown in Figure 3, after rebinding the CVs of HTHP-MIPs showed almost symmetric peaks with equal heights for the reduction and the oxidation peaks which indicates that the surface concentration of electroactive HTHP in an oxidized and reduced state is very similar and the protein is not desorbed during the reduction-oxidation cycles. The peak separation ΔEp was 45 mV at scan rates of 100 mV·s^−1^. In the overall range of the scan rates, neither anodic nor cathodic peak currents increase linearly with increasing scan rates or with the square root of scan rates which means that neither a purely surface-controlled nor a purely diffusion-controlled electrode process took place.

### 3.3. Concentration Dependence of Rebinding of HTHP to MIPs and NIPs

**Figure 4:**
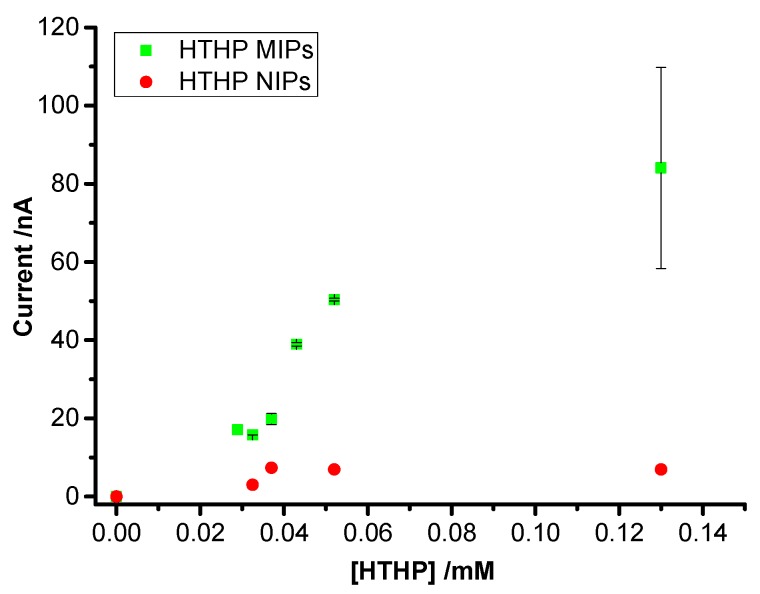
SWV peak currents for MIP- and NIP-modified electrodes after incubation in 2.5 mM K_2_HPO_4_–KH_2_PO_4_ pH 7 and different HTHP concentrations.

Rebinding of HTHP to the MIPs and NIPs after template removal was investigated by SWV. After incubation of the MIPs in a series of HTHP solutions, the redox peaks of HTHP reappeared and the peak currents increased with increasing concentration of HTHP starting from approximately 30 µM and approached saturation above 100 µM (Figure 4). Current signal increased linearly with increasing concentration. On the other hand the peak currents for the NIPs are much smaller and almost constant within this concentration range. The signal for NIPs should be attributed to “non-specific” pores in the NIP film which allows the protein to reach the MUA-SAM.

The evaluation of DET quantifies the “productively” rebound target molecules which might be different from the total amount of target bound to the MIP or NIP. In this respect, it is more specific than the measurement of the “total material bound” by quartz crystal balance or surface plasmon resonance or the very indirect measurement of the permeation of a redox marker by CV or impedance spectroscopy. The ratio of the DET signals at MIP and NIP—which represents a “functional” imprinting factor (IF)—was calculated to be 12 ± 3 at saturation. This clearly shows the higher efficiency of “productive” binding of the target protein to the imprinted film in relation to the NIP. Using the same polymer, the IF value for the cyt *c*-MIP on a MUA-SAM was 2 [19], and 8.6 for ConA on a mannose terminated SAM (Table 1) [22]. The lower value for the cyt *c*-MIP is caused by the relatively high signal for the DET at the NIP. Obviously, the harsh treatment with 1 M sulfuric acid which was applied for the removal of the target cyt *c* generated defects in the polymer layer. On the other hand, the hexameric HTHP was efficiently extracted from the MIP by incubation in glycine-HCl (pH 2.2) probably via the dissociation into subunits of the hexameric protein. This treatment did not affect the HTHP signal at the NIP. Table 1 gives an overview on the literature of electrochemically prepared protein-MIPs. It shows that the majority of papers use a redox marker for the detection of target binding. Our paper describes DET and bioelectrocatalysis of the target which is bound to the MIP. Among the few papers with DET of the target it is the first MIP for an oligomeric enzyme. The IF of the HTHP-MIP belongs to the highest values of protein-MIPs and indicates the remarkably higher electroactive surface concentration of HTHP for the MIP as compared with the NIP.

### 3.4. Binding of Cyt *c* to the HTHP-MIP

In order to check whether the MIP would preferentially bind its target HTHP, the binding of HTHP and the positively charged cyt *c* was studied on both MIP and MUA-SAM modified electrodes at low ionic strength. After incubation of the MIPs in 2.5 mM K_2_HPO_4_-KH_2_PO_4_ containing 32.5 µM HTHP, the SWV signal was well pronounced. It was almost 13 times higher than that of the MUA-modified gold electrode (Figure S4). On the other hand, the peak current for cyt *c* was almost 3.5 times smaller at the MIP/MUA electrode compared with that at the MUA-covered surface. These results show a preferential binding of the (negatively charged) HTHP to the MIP-covered SAM whilst the positively charged cyt *c* interacts more effectively with the negatively charged SAM.

The peak current for cyt *c* at the MIP-covered electrode is about 5 times smaller than the value for HTHP whilst at the bare MUA-SAM it is 8 times bigger than that for HTHP. The different influence of the SAM and the MIP layer may be caused by the fact that HTHP binds via the neutral side of the “disc” only weakly to the MUA [14] whilst cyt *c* is electrostatically bound via the positively charged lysine residues of the heme surrounding [38,39]. On the other hand, the MIP layer interacts favourably with its target HTHP as compared with cyt *c*. In spite of the smaller diameter of cyt *c* the target HTHP reaches the MUA-SAM more effectively which ensures the productive orientation for the DET.

### 3.5. Electrocatalysis of Hydrogen Peroxide Reduction

**Figure 5:**
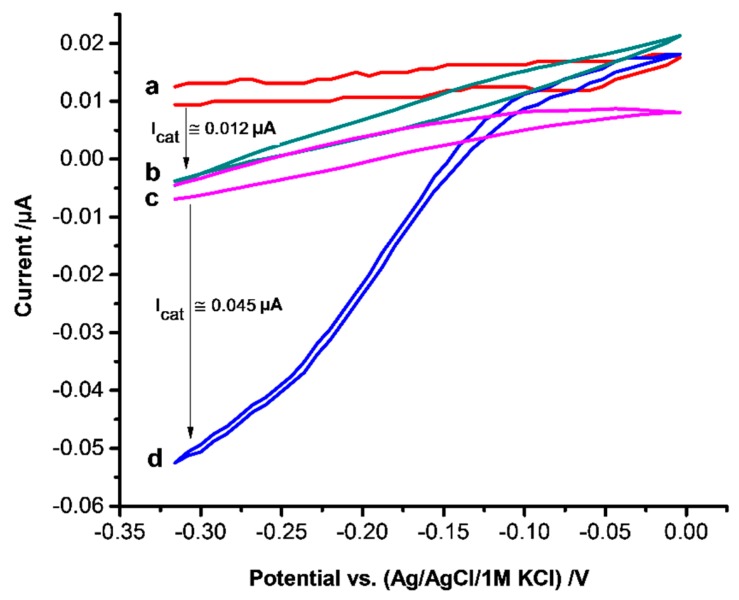
CVs of NIP (a, b) and HTHP loaded MIP (c, d) in absence (a, c) and in presence of 100 µM H_2_O_2_ (b, d) in 2.5 mM Tris buffer at pH 8, scan rate of 5 mV/s. MIP and NIP were incubated in 52 µM HTHP solution for 1 h before measurement.

The bioelectrocatalytic reduction of H_2_O_2_ by HTHP MIPs was studied by incubating the electrodes in 52 µM HTHP solution for 1 h after the removal step. As shown in Figure 5, with curve d the catalytic current starts from around −0.1 V upon addition of hydrogen peroxide. Since no mediator is present, the reduction current should arise from the DET between the electrode and the heme protein [40,41]. However, the potential is 650 mV more negative than expected for the reduction of Fe^4+^ = O^−^ center of Compound I [42] but in accordance with reports on heme protein-modified electrodes describing the peroxide reduction at the potential where the heme is in the reduced state [43,44]. The catalytic current increased linearly with increasing concentration of H_2_O_2_ in the range from 10 to 100 µM and reached saturation at 150 µM. This behavior is comparable with the effect of other heme proteins on the reduction of H_2_O_2_ [40,44,45]. NIPs which were not in contact with HTHP gave a H_2_O_2_ reduction current (Figure 5b) which is almost 3.8 times smaller than that of the HTHP MIPs.

Furthermore the peroxidatic activity of the HTHP-MIP towards NADH in the presence of H_2_O_2_ was studied. However, addition of 100 µM NADH did not cause a measurable change in the H_2_O_2_ reduction signal. This result might be explained by the low specific activity of 3 Units/mg of HTHP [20].

## 4. Conclusions

MIPs for the recognition of the hexameric heme protein HTHP with both DET and bioelectrocatalytic activity have been prepared by electropolymerization of scopoletin after oriented assembly of the target on a negatively charged SAM. Rebinding of the target to the MIP sums up the electrostatic attraction of the protein by the SAM with the shape recognition by non-covalent interactions with the MIP. This is reflected by the higher electroactive surface concentration of HTHP on the MIP covered SAM as compared with the SAM-modified electrode.

The evaluation of DET or bioelectrocatalysis quantifies the “productively” rebound target molecules. Therefore, it is more specific than the measurement of the total amount of bound material by quartz crystal balance or surface plasmon resonance or the indirect measurement of the permeation of a redox marker by cyclic voltammetry or impedance spectroscopy (Table 1). This principle is an efficient tool to develop MIPs for the almost 40 electroactive proteins described in the literature which will allow the regeneration of enzymes in sensors or fuel cells.

## Figures and Tables

**Table 1:** Examples of protein MIPs prepared by electropolymerization.

Monomer	Template/Protein	IF	Detection of Binding	Ref.
**Scopoletin**	Cyt *c*-derived peptide	6	Fluorescence, SPR	[26]
**Scopoletin**	Cyt *c*	2	Fluorescence, CV (DET)	[19]
**Scopoletin**	ConA	8.6	QCM, SPS	[22]
**Scopoletin**	Ferritin	13	SPR, AFM	[27]
**Scopoletin**	HTHP	12	CV (DET, RM), SWV	This work
**Pyrrole**	*gp51*	9–10	PAD (RM)	[28]
**Pyrrole**	Bovine hemoglobin	-	DPV (RM), EIS, SEM	[29]
**Pyrrole**	Bovine hemoglobin	7.72	DPV (RM), EIS, SEM	[30]
**3,4-Ethylenedioxythiophene/Poly(styrenesulphonate)**	Avidin	6.5	QCM, AFM	[31]
**3,4-Ethylenedioxythiophene/Poly(styrenesulphonate)**	Avidin or Av-FITC	-	Fluorescence, SEM	[32]
***o*-Phenylenediamine**	Troponin T	-	CV (RM), DPV, AFM	[33]
**Phenol**	Human ferritin/Human papillomavirus	-	EIS, DPV (RM), SEM	[34]
**3-Aminophenylboronic acid**	BSA	2	SPR, SEM	[35]
**3,4-Propylenedioxythiophene carboxylate**	Acetylcholinesterase	9.9	Amperometry of catalysis, AFM	[36]
**2,2’-Bithiophene-5-carboxylic acid**	Human serum albumin	26.8	DPV (RM), EIS, AFM	[37]

QCM: quartz crystal microbalance, SPS: surface plasmon spectroscopy, RM: redox marker, *gp51*: bovine leukemia virus glycoprotein *gp51*, PAD: pulsed amperometric detection, DPV: differential pulse voltammetry, EIS: electrochemical impedance spectroscopy, SEM: scanning electron microscopy, Av-FITC: avidin-fluorescein isothiocyanate, BSA: bovine serum albumin.

## Supplementary Materials

The following are available online at http://www.mdpi.com/1424-8220/16/3/272/s1, Figure S1: CVs of the (**a**) SAM covered Au electrode and (**b**) after incubation in 1.3 mM HTHP solution for 1 h under semi-anaerobic condition in 10 mM K_2_HPO_4_–KH_2_PO_4_, pH 8, 100 mV/s, Figure S2: CVs of the redox marker [Ru(NH_3_)_6_]^2+^ for the different steps of MIP and NIP preparation (5 mM [Ru(NH_3_)_6_]^2+^ in 10 mM K_2_HPO_4_–KH_2_PO_4_, pH 8, 100 mV/s): a—bare Au wire; b—after SAM-formation; c_MIP_—after electropolymerization in presence of the template HTHP; c_NIP_—after electropolymerization in absence of the template HTHP; d_MIP_—after removal of HTHP; d_NIP_—after removal procedure applied to NIP, e—after rebinding in 1.3 mM HTHP solution for 1 h, Figure S3: CVs of the MIP covered Au electrode under semi-anaerobic condition in 10 mM K_2_HPO_4_–KH_2_PO_4_, pH 8, 400 mV/s. (**a**) after electropolymerization; (**b**) after removal of HTHP; (**c**) after rebinding in 1.3 mM HTHP solution for 1 h, Figure S4: Normalized current signal from SWVs of (**a**) MUA/Au (set to 1) and (**b**) MIPs incubated in 32.5 µM HTHP solution for 1 h 2.5 mM K_2_HPO_4_–KH_2_PO_4_ at pH 7.

## Abbreviations

The following abbreviations are used in this manuscript:

AFM: Atomic force microscopy
Av-FITC: Avidin-fluorescein isothiocyanate
BSA: Bovine serum albumin
ConA: Concanavilin A
CV: Cyclic voltammetry
Cyt *c*: Cytochrome *c*
DET: Direct electron transfer
DPV: Differential pulse voltammetry
EIS: Electrochemical impedance spectroscopy
*gp51*: Bovine leukemia virus glycoprotein *gp51*
HTHP: Hexameric tyrosine-coordinated heme protein
IF: Imprinting factor
MIP: Molecularly imprinted polymer
MUA: Mercaptoundecanoic acid
NIP: Non-imprinted Polymer
PAD: Pulsed amperometric detection
PB: Phosphate buffer
QCM: Quartz crystal microbalance
SAM: Self-assembled monolayer
SDS-PAGE: Sodium dodecyl sulfate polyacrylamide gel electrophoresis
SEM: Scanning electron microscope
SPR: Surface plasmon resonance
SPS: Surface plasmon spectroscopy
SWV: Square wave voltammetry
