# Supplementary material for: Molecularly Imprinted Electropolymer for a Hexameric Heme Protein with Direct Electron Transfer and Peroxide Electrocatalysis
Source: Sensors (Basel). 2016 Feb 23;16(3):272. doi: 10.3390/s16030272 (PMC4813847; doi:10.3390/s16030272)
Supplement: Supplementary file 1 [file sensors-16-00272-s001.pdf]

Supplementary Materials: Molecularly Imprinted Electropolymer for a Hexameric Heme Protein with Direct Electron Transfer and Peroxide Electrocatalysis

Lei Peng, Aysu Yarman, Katharina J. Jetzschmann, Jae-Hun Jeoung, Daniel Schad, Holger Dobbek, Ulla Wollenberger and Frieder W. Scheller

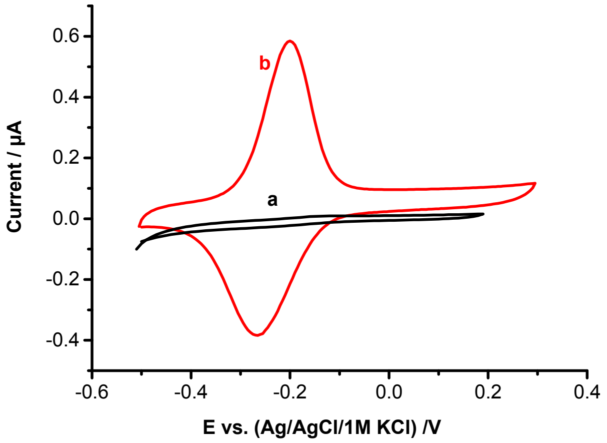

Figure S1. CVs of the (**a**) SAM covered Au electrode and (**b**) after incubation in 1.3 mM HTHP solution for 1 h under semi-anaerobic condition in 10 mM K_2HPO_4–KH_2PO_4, pH 8, 100 mV/s.

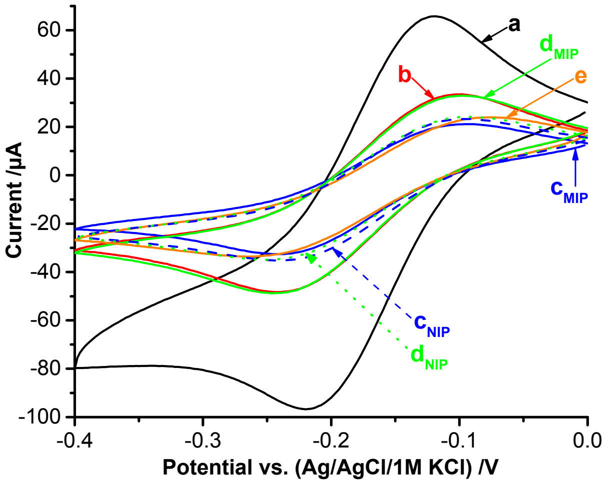

Figure S2. CVs of the redox marker $[Ru(NH_3)_6]^{2+}$ for the different steps of MIP and NIP preparation (5 mM $[Ru(NH_3)_6]^{2+}$ in 10 mM K_2HPO_4–KH_2PO_4, pH 8, 100 mV/s): a—bare Au wire; b—after SAM-formation; c_{MIP}—after electropolymerization in presence of the template HTHP; c_{NIP}—after electropolymerization in absence of the template HTHP; d_{MIP}—after removal of HTHP; d_{NIP}—after removal procedure applied to NIP, e—after rebinding in 1.3 mM HTHP solution for 1 h.

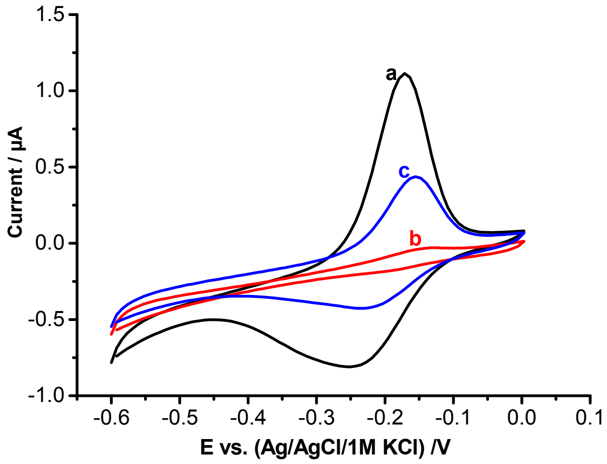

Figure S3. CVs of the MIP covered Au electrode under semi-anaerobic condition in 10 mM K_2HPO_4–KH_2PO_4, pH 8, 400 mV/s. (**a**) after electropolymerization; (**b**) after removal of HTHP; (**c**) after rebinding in 1.3 mM HTHP solution for 1 h.

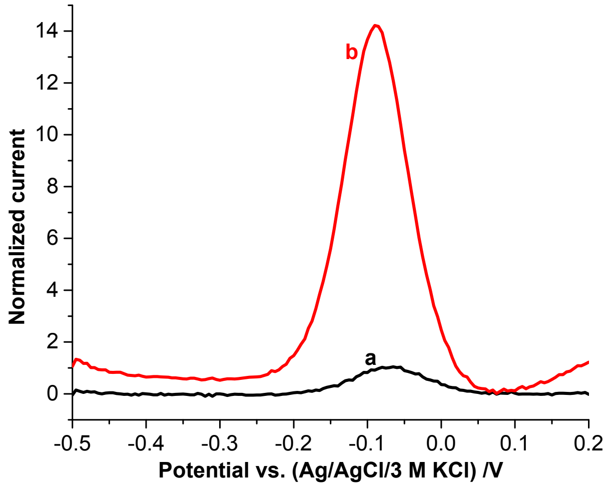

Figure S4. Normalized current signal from SWVs of (**a**) MUA/Au (set to 1) and (**b**) MIPs incubated in 32.5 µM HTHP solution for 1 h 2.5 mM K_2HPO_4–KH_2PO_4 at pH 7.
